# Genomics of asthma, allergy and chronic rhinosinusitis: novel concepts and relevance in airway mucosa

**DOI:** 10.1186/s13601-020-00347-6

**Published:** 2020-10-28

**Authors:** Anu Laulajainen‐Hongisto, Annina Lyly, Tanzeela Hanif, Kishor Dhaygude, Matti Kankainen, Risto Renkonen, Kati Donner, Pirkko Mattila, Tuomas Jartti, Jean Bousquet, Paula Kauppi, Sanna Toppila‐Salmi

**Affiliations:** ^1^ Department of Otorhinolaryngology–Head and Neck Surgery University of Helsinki and Helsinki University Hospital P.O.Box 263 Kasarmikatu 11‐13 00029 HUS Helsinki Finland; ^2^ Laboratory of Cellular and Molecular Immunology Institute of Microbiology of the Czech Academy of Sciences Prague Czech Republic; ^3^ Skin and Allergy Hospital University of Helsinki and Helsinki University Hospital Helsinki Finland; ^4^ Haartman Institute University of Helsinki Helsinki Finland; ^5^ HUS Diagnostic Center Helsinki University Hospital Helsinki Finland; ^6^ Hematology Research Unit Helsinki Department of Hematology Helsinki University Hospital Comprehensive Cancer Center Helsinki Finland; ^7^ Translational Immunology Research Program and Department of Clinical Chemistry University of Helsinki Helsinki Finland; ^8^ Department of Pediatrics and Adolescent Medicine Turku University Hospital and University of Turku Turku Finland; ^9^ Université Montpellier Montpellier France; ^10^ MACVIA‐France Montpellier France; ^11^ Corporate Member of Freie Universität Berlin Humboldt‐Universität Zu Berlin Berlin Institute of Health Comprehensive Allergy Center Department of Dermatology and Allergy Charité–Universitätsmedizin Berlin Berlin Germany

**Keywords:** Asthma, Allergic rhinitis, Airway epithelium, GWAS, Gene ontology, Pathway

## Abstract

Genome wide association studies (GWASs) have revealed several airway disease‐associated risk loci. Their role in the onset of asthma, allergic rhinitis (AR) or chronic rhinosinusitis (CRS), however, is not yet fully understood. The aim of this review is to evaluate the airway relevance of loci and genes identified in GWAS studies. GWASs were searched from databases, and a list of loci associating significantly (p < 10^–8^ ) with asthma, AR and CRS was created. This yielded a total of 267 significantly asthma/AR–associated loci from 31 GWASs. No significant CRS ‐associated loci were found in this search. A total of 170 protein coding genes were connected to these loci. Of these, 76/170 (44%) showed bronchial epithelial protein expression in stained microscopic figures of Human Protein Atlas (HPA), and 61/170 (36%) had a literature report of having airway epithelial function. Gene ontology (GO) and Kyoto Encyclopedia of Genes and Genomes (KEGG) annotation analyses were performed, and 19 functional protein categories were found as significantly (p < 0.05) enriched among these genes. These were related to cytokine production, cell activation and adaptive immune response, and all were strongly connected in network analysis. We also identified 15 protein pathways that were significantly (p < 0.05) enriched in these genes, related to T‐helper cell differentiation, virus infection, JAK‐STAT signaling pathway, and asthma. A third of GWAS‐level risk loci genes of asthma or AR seemed to have airway epithelial functions according to our database and literature searches. In addition, many of the risk loci genes were immunity related. Some risk loci genes also related to metabolism, neuro‐musculoskeletal or other functions. Functions overlapped and formed a strong network in our pathway analyses and are worth future studies of biomarker and therapeutics.

## Background

Asthma, allergic rhinitis (AR) and chronic rhinosinusitis (CRS) are multifactorial chronic airway diseases that share some common pathogenetic mechanisms. AR is caused by allergen binding to specific IgE in the nasal mucosa of a sensitized individual, leading to inflammation and symptoms of allergy. The prevalence of AR has increased in the Western countries over the last few decades and it nowadays has been estimated to affect up to 10–25% of the population [1 ]. Asthma is a chronic pulmonary disease with airway inflammation, bronchial hyperresponsiveness and recurrent, reversible airflow obstruction. Exacerbations are common both in asthma and in CRS, which is a chronic symptomatic inflammation of the sinonasal tract. Asthma and CRS both affect about 3–10% of the Western population [2., 3., 4. ]. Risk factors for asthma, AR and CRS include genetic predisposition, other allergic diseases, infections and environmental factors including exposure to tobacco smoke and air pollution [5., 6., 7. ].

Many of the environmental risk factors in the pathogenesis of asthma and CRS are linked to disrupted interplay between epithelial barriers, particles, allergens and microbes [8, 9 ]. Type 2 biased inflammation with recruitment of eosinophils, basophils, and T‐cells, and release of cytokines is common in atopic asthma and AR [10, 11 ]. Epithelial cells are in contact with microbes, which increasingly have been shown to have a role in inflammatory diseases [12, 13 ]. Recent studies have also found that altered airway microbiome composition might be associated with asthma [14 ], seasonal AR [15., 16., 17. ], or children with rhinitis [18 ].

Genetic inheritance has been estimated to explain 25–80% of asthma risk [19 ] and up to 90% of AR risk [20 ]. The genetic predisposition of CRS seems to vary according to CRS type. Although an increased risk is associated with both types, the familial risk of CRS with nasal polyps (CRSwNP) has been found significantly higher than that of CRS without nasal polyps (CRSsNP) in a population based study conducted in Utah [21 ].

Large collaborative twin studies and GWAS projects have helped establishing genetic components for asthma, CRS and AR. Kim et al. [22 ] summarized the results of 42 GWASs of asthmatic subjects and controls and asthma‐related traits. The most replicated loci with genome‐wide significant (p < 5 × 10^–8^ ) were the cluster of genes at the 17q12–21, including *ORMDL3* (orosomucoid‐like 3), *GSDMB* (gasdermin B), and *GSDMA* (gasdermin A), specific to childhood‐onset disease. The next three loci achieving significant p‐values included loci 2q12 in the vicinity of several interleukin receptor genes, namely *IL1RL1, IL1RL2*, and *IL18R1*, a region on 5q22 that contains the mitochondrial solute carrier gene *SLC25A46* and the hemopoietic cytokine gene *TSLP* and a complex region located within the major histocompatibility locus 6p21. While these multigene loci are challenging to dissect, it is notable that *IL1RL1* encodes the receptor for IL‐33. The gene that encodes IL‐33 is separately implicated in the genetic etiology of asthma through the fifth most replicated locus on chromosome 9p24.

Like many other complex diseases, the development of asthma or AR requires genetic predisposition and appropriate timing of environmental exposures. GWASs have been able to identify and replicate several significant risk regions in large sample sets [10 ]. Among already known important asthma loci, GWASs have also revealed previously undescribed and unexpected genetic components, highlighting the method's freedom of preconceptions [23 ].

**Fig 1:**
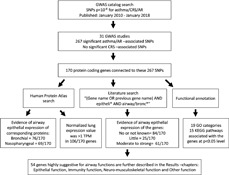
Flow chart of the study. GWASs were searched from databases (https://www.ebi.ac.uk/gwas/ ) and a list and database of SNPs associating significantly (p < 10^–8^ ) with asthma, AR and CRS was formed in 1/2018. Gene Ontology (GO) and Kyoto Encyclopedia of Genes and Genomes (KEGG) annotation analysis was performed for protein coding genes connected to SNPs. Airway epithelial expression of genes and corresponding proteins were evaluated by using Gene Cards, Human Protein Atlas (HPA), and literature search from PubMed. Other potential airway functions of the selected set of genes were evaluated from database /literature search. Abbreviations: *AR* allergic rhinitis, *CRS* chronic rhinosinusitis, *GO* Gene Ontology, *GWAS* genome‐wide association study, *HPA* Human Protein Atlas, *KEGG* Kyoto Encyclopedia of Genes and Genomes, *SNP* single nucleotide polymorphism, *TPM* Transcripts Per Million

Risk factors and pathogenetic mechanisms of allergic diseases are also interrelated and share partly same mechanisms, which is why we took this broad approach. Our aim was to review GWASs identified asthma, AR and CRS related genes, and to evaluate their relevance in airway mucosal functions. This review is based on an extensive literature search, and several database searches (Fig. 1 ).

## GWAS catalog‐search

**Fig 2:**
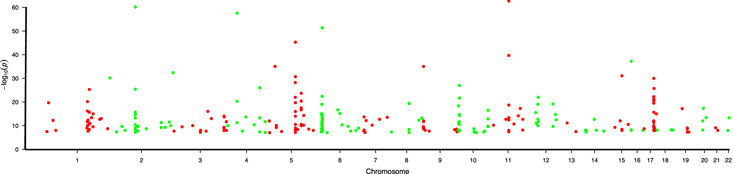
Manhattan plot (https://biorender.com/ ) of the SNPs that were significantly associated (p < 5 × 10–8) with asthma/AR in GWASs

The GWAS catalog containing 11,598 unique SNPs, was downloaded from the National Human Genome Research Institute (NHGRI) website (https://www.ebi.ac.uk/gwas/ ) on January 18, 2018. We created a list of 267 SNPs associating significantly (p < 10 exp ‐8) with asthma and/or AR provided in the Additional file 1 : Table S1. There were no SNPs associating significantly with CRS. Gene symbols were mapped onto chromosomes by using Ensemble Karyotype viewer (https://www.ensembl.org/Homo_sapiens/Location/Genome ) (Additional file 1 : Table S1). Of these 267 SNPs, we selected the SNPs which were assigned to a protein coding gene or those reported to have a protein coding gene as the nearest gene [24 ]. Using this strategy, we identified a total of 170 protein coding genes (Additional file 1 : Table S2). Of them, 21 genes were connected to several SNPs and/or identified in different studies. A Manhattan plot (https://biorender.com/ ) of SNPs was generated (Fig. 2 ), showing that susceptibility genes were distributed to all chromosomes (except sex chromosomes). Several genes were found to locate in chromosomes 1, 5, 6, or 17.

## Database and literature search of airway expression of the protein expressing genes

**Fig 3:**
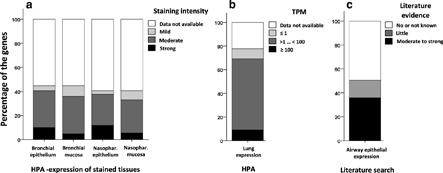
Database and literature analysis of airway expression of the corresponding proteins of the protein coding GWAS‐level genes (n = 170) associating with asthma/AR. **a** Human protein Atlas (HPA) was used to search for photomicrographs of the proteins and their staining intensity was semi‐quantitatively evaluated by two observers. **b** HPA results of lung expression in Transcripts per million (TPM). **c** Literature search results of airway epithelial expression of these genes. Pubmed search was performed by using search terms “(Gene name OR alias) AND epitheli* AND airway/bronc*”, Nasophar = Nasopharyngeal

Information about the 170 protein coding genes in Gene Cards, NCBI Gene Expression Omnibus (GEOaccession: GSE5057 and GSE40364), and Human Protein Atlas (HPA) (https://www.proteinatlas.org/ ) was examined (Fig. 3 ). We also collected lung, bronchial, and nasopharyngeal expressions of these genes from the Genotype tissue expression portal (GTEx), expressed as Reads Per Kilobase Million (RPKM) (Additional file 1 : Table S2).

Nasopharyngeal/bronchial protein expression information was obtained from immunohistochemically stained photomicrographs of HPA, and the staining intensity was semiquantitatively scored as 0–3 (0 = no, 1 = mild, 2 = moderate, 3 = strong staining). A total of 76/170 (44%) of these genes showed bronchial epithelial protein expression and 69/170 (41%) showed nasopharyngeal epithelial protein expression in stained microscopic figures (Fig. 3 a). The proportion of genes that did not have protein expression figures available was 55% in bronchial and 59% in nasopharyngeal region (Fig. 3 a). All available stained microscopic figures showed airway expression, and when scoring semiquantitatively the staining intensity, the proportion of genes showing moderate protein expression intensity of all available figures was 68% in bronchial and 64% in nasopharyngeal regions (Fig. 3 a). Similarly, the proportion of genes with overall expression was 45% in bronchial and 41% in nasopharyngeal mucosa (Fig. 3 a). When evaluating the normalized expression values, e.g. transcripts per million (TPM) in HPA, 51/170 (30%) genes did not show data, 106/170 (62%) genes showed TPM > 1 and 8% genes showed TPM value of < 1 (Fig. 3 b). The exact TPM values are shown in the Additional files 1, 2 : Tables S1 and S2. When evaluating Genotype‐Tissue Expression (GTEx) from HPA, the expression levels for the vast majority of these genes were not available (Additional files 1, 2 : Tables S1 and S2).

A systematic literature search in PubMed was performed for the 170 protein coding genes, by using as search terms “Gene name (or any of its aliases) AND epitheli* AND airway/bronc*” and information of airway epithelial expression was scored as 0–2 (0 = no evidence of airway expression, 1 = maybe, 2 = yes/ubiquitous expression) (Additional files 1, 2 : Tables S1–S2). A total of 25/170 (15%) genes showed little and 61/170 (36%) showed moderate to strong evidence of airway epithelial protein expression, whereas 49% of genes showed no/not known evidence of airway epithelial protein expression (Fig. 3 c).

## Functional annotation

**Fig 4:**
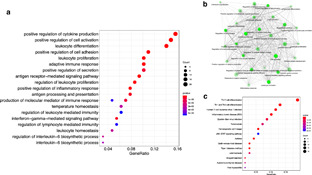
Functional annotation of the genes corresponding to the SNPs that were significantly associated with asthma/AR in GWASs. We used the whole list of SNPs (Ensemble codes) shown in the Additional file 1 : Table S1. Of these, only SNPs that were connected to protein coding genes were used in this analysis, and only one unique gene associated with one or several SNPs were used. Noncoding genes related to these asthma/AR ‐associated SNPs were excluded in functional annotation. The total number of genes of this functional annotation was 155 genes. **a** Gene ontology (GO) categories (functional protein categories) that were enriched. **b** Gene function network interaction of the GO‐categories. The network interaction shows strong interaction between the GO‐categories and indicating regulation functions between each other. **c** Kyoto Encyclopedia of Genes and Genomes (KEGG) pathways enriched among 155 genes. Shades of blue and red indicate significance of the enrichment (all were significant at level p < 0.05), and the size of the dot represents gene count. X‐axis represents the number of genes belonging to the particular category / total number of observed genes (N = 155)

We performed functional annotation of the identified genes by R, using a package called clusterProfiler (https://bioconductor.org/packages/release/bioc/html/clusterProfiler.html ) (Fig. 4 ) [25 ]. The SNPs (Additional file 1 : Table S1) that were connected to the protein coding genes were used. If several SNPs were in/near to the same gene, only the first SNP of the list of the Additional file 1 : Table S1 was used in functional annotation. Noncoding genes related to these asthma/AR ‐associated SNPs were excluded in functional annotation. Altogether, there was a total of 155 genes showing functional annotation (Fig. 4 ). We identified 19 Gene ontology (GO) categories (functional protein categories) that were significantly (p < 0.05) enriched among these genes, such as cytokine production, cell activation, leukocyte differentiation, regulation of cell adhesion, leukocyte proliferation, adaptive immune response, antigen receptor signaling pathway and regulation of inflammation response (Fig. 4 a). Gene network analysis showed strong interaction between these GO‐categories, indicating a strong regulation network between the genes (Fig. 4 b). We also identified 15 Kyoto Encyclopedia of Genes and Genomes (KEGG) pathways that were significantly (p < 0.05) enriched among the genes, such as Th17 cell differentiation, Th1 and Th2 cell differentiation, Human T‐cell leukemia virus 1 infection, Inflammatory bowel disease, Epstein Barr virus infection, Tuberculosis, Hematopoietic cell lineage, JAK‐STAT signaling pathway, and asthma (Fig. 4 c). JAK‐STAT signaling pathway was the most significantly enriched pathway (Fig. 4 c).

## Literature review

**Fig 5:**
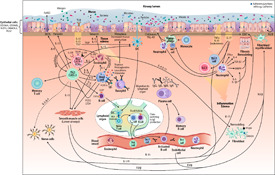
Postulated mechanisms of GWAS‐related genes in lower airways during asthma. Abbreviations: *CCR7* C‐C‐motif chemokine receptor 7, *DC* Dendritic cell, *EMT* Epithelial‐mesenchymal transition, *FCER1A* Fc fragment of IgE receptor 1A, *FeNO* Fractional exhaled nitric oxide, *FLG* Filaggrin, *GATA‐3* GATA binding protein 3, *GDSMA* Gasdermin A, *GDSMB* Gasdermin B, *HLA* Human leukocyte antigen, *IgA* Immunoglobulin A, *IgE* Immunoglobulin E, *IgG* Immunoglobulin G, *IgM* Immunoglobulin M, *IKZF3* Ikaros family zinc finger protein 3, *IL* Interleukin, *ILC2* Group 2 innate lymphoid cells, *ILC3* Group 3 innate lymphoid cells, *INFγ* Interferon gamma, *iNOS* Inducible nitric oxide synthase, *ITGβ8* Integrin Subunit Beta 8, *LTD4* Leukotriene D4, *MMP9* Matrix metalloproteinase 9, *NKT cells* Natural killer T cells, *ORMDL3* Orosomucoid‐like 3, *PGD2* Prostaglandin D2, *RORα* Retinoid‐Related Orphan Receptor Alpha, *SMAD3* SMAD family member 3 (= Mothers against decapentaplegic homolog 3), *STAT6* Signal transducer and activator of transcription 6, *TFH cell* T follicular helper cell, *TGFβ* Transforming growth factor beta, *Th1* T helper type 1, *Th2* T helper type 2, *Th17* T helper type 17, *TLR* Toll‐like receptor, *TNFα* Tumor necrosis factor alpha, *TSLP* Thymic stromal lymphopoietin, *YKL‐40* Chitinase like protein, *Remodeling* Smooth muscle and/or fibroblast proliferation, fibrosis, EMT etc.

We finally ranked genes that were highly suggestive of having asthma/AR‐relevant functions. The selection was performed based on (a.) the significance (p‐value) of particular SNP in GWASs, or (b.) multiple replication of particular SNP or gene in GWASs, or (c.) large literature knowledge of airway function of a particular gene (Additional file 1 : Table S1). The selected list of genes is shown in bold text in Table 1. We searched expression knowledge from literature for this smaller set of genes and categorized the genes based on their potential function in the airways in four main groups: genes related to epithelial function, immunity function, neuro‐musculoskeletal functions, and other functions. Although some functional groups overlap, genes are only reported in one functional group. In the following text we will discuss the most relevant genes we found in these searches. In Fig. 5 we summarize the main airway functions related to the genes we evaluated most relevant for functions in nasal mucosa during AR and in bronchial mucosa during asthma.

**Table 1:** The list of all reported genes associating with asthma/AR/CRS GWAS SNPs at the level of P < 10 exp ‐8. The reviewed genes are shown in **bold**

Chr	Genes
1	*SFPQ, ZMYM4*, ***RUNX3***, *RERE, TNFRSF14, FAM213B, C1orf54, MRPS21,* ***FLG,*** *IL6R, RORC, RPTN, HRNR,* ***PYHIN1***, *DARC*, ***FCER1A, OR10J3***, *NDUFS2, FCER1G, CD247, FASLG, TNFSF18, TNFSF4, CRB1, DENND1B*, ***CHI3L1***, *ITPKB*
2	*ASB3, SOCS, JUND, CEBPB, IL18R1*, ***IL1RL1***, *IL1RL2, BCL2L11, ANAPC1, IL1B, KYNU, ARHGAP15*, ***PLCL1***, *IKZF2, CCL20, DAW1, INPP5D, D2HGDH*
3	*RYBP, GLB1, IL5RA*, ***ABI3BP***, *FAM172B, TRMT10C, SLC15A2, GATA2, RASA2,* ***BCL6, LPP*** *, DLG1, FBXO45, CEP19*
4	***TLR1, TLR6, TLR10,*** *STX18, MSX1, SRIP1*, ***GC***, *MANBA*, ***ADAD1, IL2, IL21***, *GAB1*
5	*DAB2, PTGER4*, ***IL7R***, *FBXL7, FAM105A, PDE4D, TMEM232,* ***SLC25A46, TSLP, WDR36***, *CAMK4, TNFAIP8, C5orf56,* ***IL13, RAD50***, *IL5, DIAPH1, NDFIP1, LMAN2, RGS14*
6	*GRM4, HGMA1, ITPR3, BTNL2, C6orf10, HLA‐DPB1, HLA‐DOA, HLA‐DPA1,* ***HLA‐DQA1, HLA‐DQA2, HLA‐DQB1***, *HLA‐DRA, BTNL2, NOTCH4,* ***PBX2***, *HLA‐B, MICA, HLA‐C, NCR3, AIF1,* ***PSORS1C1, TNXB***, *CREBL1, HLA‐A, HLA‐G, HLA‐J, BACH2, ATG5, PTPRK, TNFAIP3, ARID1B, RNASET2*
7	*C7orf72, IKZF1, JAZF1, NPY, FERD3L,* ***ITGB8***, *ABCB5, GSAP*, ***CDHR3***
8	*TUSC3, ZBTB10, TPD52*, ***SLC30A8, MYC***
9	*EQTN, TEK, MOB3B, JKAMPP1, TYRP1, JAK2, RANBP6*, ***IL33***, *PHF19, TRAF1, C9orf114, LRRC8A, PTGES*
10	***GATA3***, *SFTA1P, AKR1E2, IL2RA, ZNF365, JMJD1C, REEP3, PSAP, HPSE2, C10orf95, ACTR1A, TCF7L2*
11	*DBX1, NAV2, HTATIP2, PRMT3,* ***AP5B1, OVOL1***, *WNT11,* ***LRRC32, C11orf30***, *SESN3, FAM76B, LAYN, SIK2, DDX6, CXCR5, KIRREL3‐AS3, ETS1*
12	*HDAC7*, ***AQP2***, *CDK2, SUOX, IKZF4*, ***STAT6***, *NAB2, ATXN2, SLC22A5, C12orf65, CDK2AP1, SPPL3, HNF1A‐AS1*
13	*FOXO1, PIBF1, KLF5*
14	*PSMA6, FOXA1, TTC6, RAD51B, JDP2, BATF, RCOR1, TRAF3*
15	*RTF1, ITPKA,* ***RORA, SMAD3***, *IQGAP1*
16	*CLEC16A, RMI2, LITAF*
17	*SMTNL2, ALOX15, GRB7*, ***GSDMA, GSDMB***, *CRKRS, ORMDL3, PERLD1*, ***IKZF3***, *PNMT,* ***PSMD3***, *ZPBP2*, ***CCR7, SMARCE1***, *STAT5B, MAP3K14, ARHGAP27, ZNF652*
18	*LPIN2, DYNAP, RAB27B, TNFRSF11A*
19	*SLC7A10, CEBPA,*, ***ZNF614, ZNF841, ZNF432***, *ZNF776*
20	*NFATC2*, ***ZNF217***, *RTEL1*
21	*RUNX1, SIK1*
22	*IL2RB, TEF, TOB2*

### Epithelial function—related genes

*CHI3L1* (Chitinase‐3‐like protein 1) gene encodes chitinase like protein (YKL‐40), which is involved in inflammation and tissue remodeling [26 ]. A study combining GWAS with serum YKL‐40 measurement, involving 632 members of the Hutterite population of European decent (living in South Dakota) (age range 6–92 years; asthma in 11.5%, atopy in 41.2%), have shown *CHI3L1* (SNP rs4950928, ‐131C‐ > G) to be a susceptibility gene for asthma, bronchial hyperresponsiveness, and reduced lung function [27 ]. Elevated circulating YKL‐40 levels have been shown to be a biomarker for asthma [27, 28 ], they have been shown to correlate with asthma severity, thickening of subepithelial basement membrane, and inversely with lung function [26 ].

*CDHR3* (Cadherin related family member 3) gene is a transmembrane protein with six extracellular cadherin domains [29 ]. Cadherins are highly expressed in airway epithelium, and are involved in cell adhesion, epithelial polarity, cell–cell interactions and differentiation [29 ]. *CDHR3* is expressed in FOXJ1‐expressing ciliated cells, which are also the targets of Rhinovirus C (RV‐C) binding [30 ]. GWAS was performed for blood samples of 1173 Danish children (2–6 years) with recurrent acute hospitalizations for asthma, and *CDHR3* (SNP rs6967330; p.Cys529Tyr) has been identified as a susceptibility gene for early childhood asthma with severe exacerbations [29, 31 ]. Since asthma exacerbations are often caused by infections, it is possible that *CDHR3* variations increase susceptibility to infections, and exacerbations, because of disrupted epithelial integrity [31 ].

Chromosome 17q21 is an area of interest for asthma and contains a cluster of genes linked to asthma in several GWAS studies, including the *GSDMB* (Gasdermin B) and *IKZF3* (Ikaros family zinc finger protein 3) genes [32 ]. Chromosome 17q21 has also been linked to inflammatory bowel disease, primary biliary chirrosis, and type 1 diabetes mellitus [32, 33 ].

Gasdermin A (GSDMA) and B (GDSMB) belong to a family of pore‐forming proteins, causing membrane permeabilization and pyroptosis, which is a lytic pro‐inflammatory cell death type [34 ]. Gasdermins are involved in inflammation and cell death, in several herediatry diseases, auto‐inflammatory diseases and cancer [34 ]. *GSDMB* is highly expressed in ciliated airway epithelial cells [35 ]. Associations have been shown between *GSDMA* gene and asthma [36, 37 ] and, between *GSDMB* gene and asthma [38 ] or early childhood asthma with severe exacerbations [39 ]. Moffat et al. 2010 found an independent association with childhood‐onset asthma and *GSDMA* gene (rs3894194, G‐> A) in their large consortium‐based GWAS of asthma in children and adults from several different populations [36 ]. Ferreira et al. 2014 found in their GWAS meta‐analysis including children and adults from different populations, that *GSDMA* gene (rs7212938, G) was associated with risk of asthma without hay fever (OR 1.14, 95% CI 1.07–1.22) and less with risk of hay fever without asthma (OR 1.02, 95% CI 0.98–1.06), suggesting that it is a stronger risk factor for asthma than hay fever [37 ].

The *GSDMB* gene (rs11078927, C‐> T) has been linked with asthma by Torgerson et al. 2011, in a GWAS meta‐analysis including children and adults from different populations [38 ]. Also Bønnelykke et al. 2014, linked *GSDMB* gene (rs2305480, G) with early childhood asthma with severe exacerbations in their GWAS of Danish children [39 ].

A large GWAS of European 180,129 adults/children with asthma and/or AR and/or eczema and 180,709 healthy controls showed that *GSDMB* gene (rs921650 A) is a stronger risk factor for asthma or hay fever than for eczema [40 ]. A meta‐analysis of GWASs of self‐reported pollen, dust‐mite or cat allergy of 22 012 allergic subjects and 31 850 healthy controls showed that *GSDMB* gene (rs9303280, T‐> C) was most strongly associated with asthma [41 ]. A meta‐analysis of GWAS of 2144 asthmatic Puerto Ricans and 2893 healthy controls (adults & children) found *GSDMB* gene (rs2305480, G—> A and rs11078927, C—> T) to be associated with asthma [42 ]. *GSDMB* was highly expressed in nasal epithelial brushings of Puerto Rican children [43 ], and in primary bronchial epithelium of asthmatic lung [44 ]. A Dutch GWAS included 920 physician diagnosed asthmatic subjects with bronchial hyperresponsiveness and 980 healthy controls, from northern Netherlands, both children and adults. They compared the GWAS results to prior GWASs, and also performed lung tissue eQTL analysis of the top SNPs replicated in the GWAS analysis [45 ]. The 17q21 locus achieved genomewide significance, with *GSDMB* (rs8067378, G; rs2305480, A; rs2290400, C; rs7216389, C), also in eQTL analysis, *GSDMB* showed larger effect sizes compared to prior published literature [45 ]. Exactly how gasdermins affect asthma risk remains unknown, membrane permeabilization and pyroptosis may have an effect in this [35 ]. When GSDMB protein is cleaved by inflammatory caspase‐1 to release its N‐terminal fragment, potent pyroptotic cell death had been shown to be induced in airway epithelial cells [35 ]. There is some evidence that a *GSDMB* splice variant, associated with lower asthma risk, causes an exon deletion leading to GSDMB protein losing its ability to induce pyroptosis in airway epithelial cells, possibly reducing asthma risk [35 ].

Ikaros family zinc finger protein 3 (IKZF3)s are transcriptional factors involved in lymphocyte differentiation [46 ] and are expressed in human airway epithelial cells [47 ]. In a GWAS of Dutch asthmatics with bronchial hyperresponsiveness, *IKZF3* (rs9303277, T) was one of the top findings, and also significant in the lung tissue eQTL analysis [45 ]. In the meta‐analysis of GWAS of asthma in Puerto Ricans (children and adults), the only locus that achieved genome‐wide significance for asthma risk was *IKZF3*, (rs907092) at chromosome 17q21 [42 ]. *IKZF3* ´s role in asthma pathogenesis is unknown.

Zinc finger proteins are involved in many cellular processes, and in the development and differentiation of several tissues [48., 49., 50. ]. They are involved in tumorigenesis, cancer progression and metastasis formation e.g. in breast cancer, but can also act as tumor suppressor genes, and are also involved in neurodegeneration, skin diseases (e.g. psoriasis) and diabetes mellitus [48., 49., 50. ]. In the large GWAS of broad allergic disease phenotype (asthma and/or hay fever and/or eczema) by Ferreira et al. [20 ], *ZNF217* (Zinc Finger Protein 217) gene was one of the identified loci containing genetic risk variants independently associated with the risk of allergic disease [40 ]. In a GWAS study of Caucasian asthmatic children of which 172 were treated with budesonide and 409 with placebo or nedocromil, the *ZNF432* gene (rs3752120, T—> C) variants were associated with inhaled corticosteroids modulating bronchodilatator response, also *ZNF614* (rs2288884, rs3450) and *ZNF841* (rs12460587, G‐> T, rs3450) were closely associated [51 ].

Filaggrin (FLG) is a protein that is critical for keratinization and epithelial barrier homeostasis [52 ]. Filaggrin gene defects are known to increase the risk of allergic sensitization, atopic eczema and AR [53 ]. It is the most important genetic risk factor for atopic dermatitis [52, 54 ]. A GWAS of 1563 European children with physician diagnosed asthma and 4054 controls and a replication analysis showed that, the risk for asthma caused by *FLG* variants (R501X and 2282del4) is limited to asthma cases with co‐existing atopic dermatitis [55 ]. *OVOL1* gene encodes a putative zinc finger containing transcription factor that is highly similar to homologous protein in Drosophila and mouse. *OVOL1* regulates FLG expression in atopic dermatitis subjects [56 ], and in normal human epidermal keratinocytes [57 ]. It has been suggested that *FLG* mutations might be involved in barrier dysfunction leading to e.g. asthma. However, in immunohistochemical analysis, filaggrin was not found to be expressed in normal upper airway epithelium in a disagreement with this theory [58 ].

Toll‐like receptors (TLRs) are expressed in nasal epithelium [59 ], and they have an important innate immunity function recognizing external pathogens and activating immune responses [41 ]. Nasal epithelial *TLR* gene expression levels were not remarkably altered after nasal birch pollen challenge [59 ] although a decrease in nasal epithelial TLR1 and TLR6 protein expression was detected in birch pollen allergic adults after challenge [59 ]. In the meta‐analysis of GWASs that have been performed on both children and adult populations with self‐reported allergy, the found shared susceptibility loci with asthma included (rs2101521, A‐> G) chromosome 4p14 near *TLR1*, *TLR6* and *TLR10* [41 ]. In the meta‐analysis of GWASs of children and adults with asthma and hay fever, and controls, *TLR1* (rs4833095, T) was associated with the risk of asthma with co‐existing hay fever [37 ]. The *TLR1* gene was also found to be one of the identified loci containing genetic risk variants independently associated with the risk of allergic disease in the large GWAS of broad allergic disease phenotype (asthma and/or hay fever and/or eczema) by Ferreira et al. [40 ].

SMAD family member 3 (*SMAD3* ) is a transcriptional modulator activated by TGFβ and it may regulate homeostatic and healing pathways to epithelial damage [36 ]. Mice with SMAD3 deficiency have increased amounts of proinflammatory cytokines in their lungs [60 ]. The GWAS by Moffat et al. showed an independent association between asthma and the *SMAD3* SNP (rs744910, G‐> A) [36 ]. In two GWAS meta‐analyses, *SMAD3* SNP (rs17228058, A‐> G) was a susceptibility locus of asthma and self‐reported allergy [41 ] and, asthma with co‐existing hay fever, but not asthma alone [37 ].

The role of epithelial to mesenchymal transition (EMT) has a critical role in airway remodeling. Human eosinophils co‐cultured with bronchial epithelial cells induced EMT, suggestive of their role in airway remodeling, with increased expression of TGFβ1 and *SMAD3* phosphorylation in the bronchial epithelial cells [61 ].

*ITGB8* gene encodes Integrin Subunit Beta 8. This protein noncovalently binds to an alpha subunit to form a heterodimeric integrin complex. In general, integrin complexes mediate cell–cell and cell‐extracellular matrix interactions and this complex plays a role in human airway epithelial proliferation. High expression levels of *ITGB8* have been associated with high angiogenic and poorly invasive glioblastoma tumors. Inactivation of *ITGB8* in the murine airway has been associated with a reduction in IL‐1β–induced airway inflammation and fibrosis, which is due to decreased TGF‐β activation [62 ].

### Immunity function–related genes

Immunity related SNPs in asthma are mostly in genes linked to HLA region and type 2 inflammation. The region 6p21 (*HLA* region) is one of the most replicated asthma loci [22 ]. Several significant SNPs have been associated with class II major histocompatibility antigen (*HLA‐DR* ) genes including *HLA‐DQA1*, *HLA‐DQA2* and *HLA‐DQB1* [38 ]. They play a central role in the immune system by presenting peptides derived from extracellular proteins. Class II molecules are expressed in antigen presenting cells, ie. B lymphocytes, dendritic cells and macrophages and are extensively studied because of the association with several autoimmune, infectious and inflammatory diseases [63 ].

Group‐specific Component (*GC* ) gene [also known as Vitamin D‐binding protein (*VDBP* ) gene] on chromosome 4q13 and has been found to associate with asthma in children [64 ]. The rs7041 G‐allele was found with increased risk [OR 2.15, CI 95% (1.32–3.50; P = 0.002)] of asthma in codominant, dominant, recessive and allelic models [64 ]. VDBP carries circulating vitamin D to the target organs, it is a chemotactic factor for leukocytes and macrophage activation, and also has a role in osteoclast activation [64, 65 ].

Approximately half of the patients with asthma, regardless of the severity of the disease, exhibit type 2 endotype. The endotype is characterized by a predominant activation of Th2 cells that produce cytokines such as interleukins 4, 5, and 13. These interleukins are responsible of Th2 cell differentiation, maturation and release of eosinophils and proliferation of IgE‐producing B‐cells, respectively [66 ]. IL‐25, IL‐33 and TSLP are thought to be master regulators of type 2 inflammation in diseases and they can all activate innate and adaptive immune cells to secrete IL‐5 and IL‐13 [67 ].

*Interleukin 1 receptor like 1* (*IL1RL1, ST2* ) is an important asthma gene and part of a cytokine receptor gene cluster. GWASs have reproducibly found the *IL1RL1* gene to be associated with asthma susceptibility [22 ]. *IL1RL1* encodes different isoforms of the receptor: IL1RL1‐a is a soluble form and IL1RL1‐b is a transmembrane receptor. IL1RL1‐a functions as a decoy receptor to dampen IL‐33 induced signaling. IL1RL1‐b together with IL1RAcP forms a heterodimeric transmembrane receptor for its ligand, IL‐33. Binding of IL‐33 initiates an MyD‐88‐mediated signaling cascade, releasing pro‐inflammatory cytokines IL‐4, IL‐5 and IL‐13 [68 ]. *IL‐33‐ST2* (*IL‐1RL1* ) axis has been regarded as one of the key players also in allergic diseases, asthma and atopic dermatitis [69 ]. A number of studies have indicated that IL‐33 induces the activation and expansion of group 2 innate lymphoid cells (ILC2s) which cause allergic inflammation by producing large amounts of IL‐5 and IL‐13 [70 ]. As IL‐5 is the main component of eosinophil activation and survival, anti‐IL‐5 treatments are used to inhibit eosinophilic inflammation. At the moment, three biologics targeting IL‐5 signaling are available: mepolizumab and reslizumab, which bind to IL‐5 directly reducing the production and survival of eosinophils, and benralizumab, which targets the IL‐5 receptor expressed on eosinophils causing a direct destruction of the cell type [71 ]. Many functional studies of asthma have focused on peripheral blood mononuclear cells, yet there are also some studies on granulocyte functions: a study has showed that persistent high blood neutrophilia was associated with poor asthma control [72 ].

Region 9p24 also belongs to one of the most replicated asthma loci, associating with the *IL‐33* gene. Being one of the major upstream regulators of type 2 inflammation, *IL‐33* has been linked to both asthma and allergic inflammation. It also functions as an”alarmin” and is secreted following tissue damage caused for example by an infection [68 ]. *IL‐33* expression in the lungs is increased in asthma [73 ]. A recent publication describes a rare loss‐of‐function mutation in *IL‐33,* protecting from asthma [74 ]. Recently, a biologic recombinant protein called IL‐33trap was shown to neutralize IL‐33 and inhibit acute allergic airway inflammation in a mouse model [75 ]. Clinical trials are ongoing with several monoclonal antibodies targeting *IL‐33/ST2* signaling [76 ].

IL‐13 is a cytokine secreted by activated Th2 cells, and acts as an important mediator of allergic inflammation pathogenesis. It shares a common receptor subunit with IL‐4, namely IL‐4Ralpha, therefore sharing also many functions with it, including promoting B‐cell proliferation and class switch to IgG4 and IgE [77 ]. Dupilumab, a biologic targeting this common receptor subunit, has been shown to be effective in many allergic diseases including asthma, atopic dermatitis [78 ] and CRSwNP [79 ]. Some functions of IL‐13 are independent of IL‐4, and especially mucus hypersecretion, subepithelial fibrosis and stimulation of matrix metalloproteinases resulting in emphysematous changes in mouse model has been shown to result from the functions of IL‐13 [80, 81 ]. A recent study investigated the association of 236 candidate gene polymorphisms and asthma disease severity, and found only one marker, the rs848 in the *IL‐13* gene region, significantly associating with symptom severity in adults with asthma [82 ].

*TSLP* (thymic stromal lymphopoietin) gene encodes a cytokine that is expressed mainly in epithelial cells and plays a key role in allergic inflammatory responses [83 ]. In humans, dendritic cells are the major target for TSLP, having an integral role on promoting Th2 cell responses. TLSP is produced by the airway epithelium in response to inhaled allergens and proinflammatory stressors and has an upstream role in the asthma cascade [83 ]. Tezepelumab binds to TSLP, inhibiting its stimulating activity on dendritic cells and innate lymphoid cells thus preventing the induction of type 2 cytokines IL‐4, IL‐5 and IL‐13. Anti‐TSLP treatment with tezepelumab decreased asthma exacerbations significantly [84 ] and phase 3 trials are ongoing.

The expression and production of Th2 cytokines IL‐4, ‐5 and ‐13, have been shown in isolated cell systems and invertebrates to be controlled by the zinc finger transcription factor *GATA3*, which is essential for Th2‐cell differentiation and activation and is considered to be the master transcription factor of the Th2 pathway of immune activation [85 ]. GATA3^+^ Th2 cells have been observed in specimens from bronchoalveolar lavage and lung biopsies obtained from patients with severe asthma, even after continuous per oral corticosteroid [86 ]. CD2‐Gata3 transgenic mice developed allergic airway inflammation and showed enhanced levels of IL‐5 and IL‐13 in bronchoalveolar lavage and lung tissue after allergen induction [87 ]. A novel therapy for the treatment of Th2‐driven asthma targeted GATA3, a transcription factor that plays a key role in Th2 cell differentiation, through an inhaled DNA enzyme (DNAzyme) that specifically cleaves and inactivates *GATA3* mRNA. In a study of allergic asthmatic patients with sputum eosinophilia and biphasic early and late asthmatic responses after allergen provocation, inhalation of GATA3‐specific DNAzyme once daily for 28 days attenuated both early and late asthmatic responses to allergen provocation when compared with placebo [88 ].

The protein encoded by *STAT6* gene is a member of the signal transducer and activator of transcription (STAT) family of transcription factors. In response to cytokines IL‐4 and IL‐13, STAT6 is phosphorylated by the receptor associated kinases, and then form homo‐ or heterodimers that translocate to the cell nucleus where they act as transcription activators for a large number of genes involved in macrophage polarization [89 ]. STAT6 has been demonstrated to regulate many characteristic features of lung inflammation common in asthma, including airway eosinophilia, epithelial mucus production, Th2 cell differentiation, and IgE production from B cells [90 ].

On chromosome 17q21, two intergenic variant SNPs between *CCR7* and *SMARCE1* associate with high p‐value in GWAS reported by Ferreira et al. [40 ]. As mentioned above, the locus 17q21 is the most replicated in asthma GWASs, including many other important asthma genes such as *ORDLM3* and *GSDMB* [22 ]. The c–c‐motif chemokine receptor CCR7 is a member of the G protein‐coupled receptor family. It is responsible for the proper recruitment of lymphocytes and mature dendritic cells to lymphoid tissues. Dendritic cells, T‐lymphocytes and B‐lymphocytes express CCR7 on their surface, and it has been shown to promote the internalization of antigens by DCs, and to regulate cell survival, migration, and to induce dendritic cell maturation [91, 92 ]. Recent study investigated the effects of *CCR7* knockdown and overexpression on dendritic cell‐mediated immune tolerance in the lungs of rats with allergic asthma and found that CCR7 expression levels affected the expression of various cytokines such as IL‐12, IL‐4, IFN‐γ and IgE, as well as the amount of immune cells in the lungs [93 ].

*FCER1A* (Fc fragment of IgE receptor 1A), an initiator of the allergic response, is located on chromosome 1q23, next to *OR10J3* coding for an olfactory receptor, and showing more evidence on association. Variants in *FCER1A* has been reported to associate with total IgE levels, allergic sensitization [94 ] and atopy [95 ]. Association of *FCER1A* polymorphism with CRSwNP has been studied in North Indian population‐based case–control study [96 ]. Although no significant association was found with CRSwNP alone, a significant association (P < 0.05) of rs2427827 SNP with high IgE level CRSwNP patients was revealed [96 ]. FCER1A has been shown to be expressed in mast cells and basophils as well as in monocytes and dendritic cells, and it has been suggested to have a dual role in IgE‐signaling – studies conducted using transgenic mouse models have shown that on one hand, FCER1A expression induces type 2 inflammation in the lungs after viral infection, on the other hand it has been linked to regulatory role in asthma, promoting immune homeostasis (reviewed in [97 ]).

*ADAD1—IL‐2/IL‐21* : Hinds et al. report a SNP rs4145717‐T in the 4q27 region that falls in the *ADAD1* gene, but the nearby *IL‐2* and *IL‐21* genes show more evidence on association [41 ]. In this region is another SNP associated with allergic rhinitis [98 ]. The IL‐2 and IL‐21 cytokines are involved in the regulation of multiple helper T cell types: IL‐21 is needed for germinal center formation by generation of T follicular helper cells [99 ] and IL‐2 is required for Th1, Th2 and Th17 cell differentiation [100 ].

### Neuro‐musculoskeletal function–related genes

*C11orf30‐LRRC32* region has been associated with asthma in previous studies [22 ]. *LRRC32* (also known as *GARP*, glycoprotein A repetitions predominant) (11q13) is expressed especially in lung and placenta and considered to be involved in several processes. It is a surface molecule of T regulatory cells [101 ] and earlier found in association to atopic dermatitis and induction of tolerance. C11orf30/EMSY is a transcriptional factor associating with tumor suppressor BRCA2, and it has been linked to above‐mentioned TSLP activation in eosinophilic esophagitis [102 ]. In a multicenter population‐based study, *C11orf30* ‐rs2155219 was reported to double the risk of polysensitisation [103 ]. Polysensitisation is associated to asthma [104 ]. *C11orf30/EMSY* has also been found a risk locus for both peanut and food allergy [105 ].

*MYC* encodes a transcription factor and located in the chromosome 8q24. MYC is a proto‐oncogene and involved in Burkitt lymphoma and multiple myeloma and serves as a prognostic factor in acute myeloid leukemia [106 ]. A study showed that asthmatics have increased MYC expression in peripheral blood ILC [107 ]. They deleted *c‐Myc* from murine lung ILC2 or an ILC2 cell line by CRISPR knockout, and showed reduced proliferation, decreased cytokine production, and reduced expression of many lymphocyte activation genes. In murine model of airway epithelial injury, *Myc* regulated proliferation and Fibroblast growth factor expression in airway smooth muscle [108 ].

Chromosomal area 6p21 (within the major histocompatibility complex gene) also includes Tenascin XB gene (*TNXB)* which is a member of tenascin family and extracellular matrix glycoproteins, and has musculoskeletal functions [55 ]. Tenascins are considered to be anti‐adhesive and associated to wound healing, they have also been associated to Ehlers‐Danlos syndrome and to malignant mesothelioma and are considered to be involved in airway remodeling in asthma [109 ]. Also the pre‐B‐cell leukemia homeobox 2 (*PBX2* ) gene (rs204993), located in 6p21, is associated to both asthma and AR in Chinese population [110 ]. *PBX2* is expressed particularly in epithelium [110 ].

*PSMD3* (chromosome 17q21) is a multicatalytic proteinase complex for proteasome and are distributed in many cells and have been reported to associate to atopic march and to atopic eczema and wheeze [111 ].

*RAD50* (chromosome 5q31) is expressed in many tissues and needed for DNA double‐strand break repair and other activities essential for cell growth. Further, it has been reported to associate with atopic dermatitis in Korean population [112 ]. *RAD50* has been shown to be expressed both in bronchial epithelial cells and bronchial alveolar lavage. However, *IL13*, locating in the same chromosomal region, showed more evidence on association with asthma [113 ].

*RORA* (chromosome 15q) is expressed especially in skin and adrenal gland and to less extent other tissues and is a member of NR1 subfamily of nuclear hormone receptors interacting in organogenesis and circadian rhythm [114 ]. Seven *RORA* SNPs were associated with childhood asthma in European populations, and *RORA* show epistasis with *NPSR1* [115 ]. The group showed in cell models that stimulation of NPSR1 activated RORA‐relevant pathway [115 ], and that NPS induced *RORA* mRNA expression in neuroblast cell line [116 ].

The *RUNX* gene family encodes Runt‐related transcription factors RUNX 1, 2 and 3. *RUNX3* (1p36) is expressed especially in bone marrow, lymph nodes and spleen and is involved in activating or suppressing transcription and is associated to tumor suppressor. *RUNX3* has been found to be hypomethylated and with increased association to inner city asthma in children [117 ]. RUNX1 is associated with aberrant B cell maturation and is related to acute myeloid or chronic leukemia [118 ]. Our study group showed that association between maternal smoking exposure and incident asthma in adult offspring was accentuated in offspring who had haplotype rs11702779‐AA of *RUNX1* gene [119 ].

*WDR36* (chromosome 5q22) encodes a member of the WD repeat protein family, involved in many cell functions e.g. signal transduction and apoptosis, and it has been associated with asthma and allergy [120., 121., 122. ]. In recent candidate gene analysis in Han Chinese population, it has been shown to associate with AR [123 ]. In vitro‐ studies show the involvement of WDR36 in G_q_ ‐coupled muscarine, bradykinin and histamine receptor signaling [124 ], all of which are important modulators of allergic reactions and asthmatic bronchoconstriction.

### Other function–related genes

The Solute Carrier family members belong to the mitochondrial transporter family, which have an important role in metabolism [125 ]. The tissue distribution and cellular/subcellular expression of *SLC25A46* (Solute Carrier family 25 member 46) is ubiquitous [125 ]. A GWAS meta‐analysis of 3933 European adults with allergic rhinitis and 8965 controls, and 2315 subjects with grass sensitization and 10 032 controls showed that a variant (rs2155219) located close to *SLC25A46* was associated with AR/grass sensitization [98 ]. The large GWAS of broad allergic disease phenotype (asthma and/or hay fever and/or eczema) by Ferreira et al. [40 ] identified also *SLC25A46* as one of the identified loci containing genetic risk variants independently associated with the risk of allergic diseas. A GWAS including Japanese and Korean pediatric asthma patients and controls, identified *SLC30A8* (Solute Carrier Family 30 member 8) SNP (rs3019885—> T/G) to associate strongly with pediatric asthma. *SLC30A8* is also known as Zinc Transporter 8, it is a zinc efflux transporter, highly expressed only in the pancreas, its variants are associated with diabetes mellitus type 2 [126 ]. It is not known how the Solute Carrier family members affect the risk of asthma.

LPP (Lipoma‐preferred partner) is a member of a protein family regulating cytoskeletal organization, cell motility and mechanosensing and functions as a mediator of transforming growth factor β (TGFβ) induced cell migration and invasion in breast cancer cells [127 ]. A study group has found that the *LPP* gene (rs9860547, G‐> A) is a shared susceptibility locus of asthma and self‐reported allergy, with the risk allele being protective against allergy in the meta‐analysis of GWASs, in children and adults with self‐reported allergy and controls [41 ]. The study group also performed eQTL analysis, the results of which suggested that the *LPP* gene association may be mediated by an effect on *BCL6* (B cell lymphoma 6) expression affecting STAT6‐mediated responses on IL‐4, IL‐13, and IgE class switching [41 ].

Elevated sputum *PSORS1C1* (Psoriasis Susceptibility 1 Candidate 1) levels have been shown in chronic obstructive pulmonary disease (COPD) [128 ]. Pyrin and HIN domain family member 1 (*PYHIN1* ) is related to initiation of innate immune response via detection of foreign DNA and, PYHIN1 was shown to positively regulate LPS‐induced IFN‐β and NO production through up‐regulating the MyD88‐independent signaling pathway in murine macrophage cell model [129 ]. Aquaporins (AQPs) mediate fast transmembrane transport of water thus regulating fluid balance in the organs [130 ]. *AQP2* gene encodes kidney´s water channel protein and it mediates urine water concentration and regulates water balance [130 ]. A mouse model showed that a Chinese herb, Platycodon root, by diffusing the lung can ameliorate the respiratory‐function and pathologic changes in the lung tissues, but also regulate urinary output and renal expression of AQP1 and AQP2 [130 ].

*PLCL1* (Phospholipase C‐Like 1) is involved in an inositol phospholipid‐based intracellular signaling cascade [41 ]. It has been reported to be related to Circadian entrainment [131 ] and Crohn's disease [41 ]. ABI3BP (ABI Family Member 3 Binding Protein) is a extracellular matrix protein and is expressed in multiple organs, including the heart, kidney, lung, pancreas, and placenta, with low‐level or variable expression in the spleen, liver, brain, bone, and skeletal muscle [132 ]. *ABI3BP* gene has been shown to contribute emphysema phenotype in a mouse model that were exposed to cigarette smoke [133 ]. On the other hand a study showed that knockout of *Abi3bp* in mice does not affect their olfactory function, mental state and NNK‐induced lung tumorigenesis [134 ]. *AP5B1* (Adaptor Related Protein Complex 5 Subunit Beta 1) is associated with Hereditary Spastic Paraplegia [135 ].

## Conclusions

Development of post‐GWAS methods are important for characterizing the function of trait‐associated loci [136 ]. Strategies integrating various biological data sets with GWAS results will provide insights into the mechanistic role of associated loci. For example, an integrated GWAS and expression study on AR highlights mitochondrial pathways as a target for further investigation of AR mechanism and treatment [137 ].

We identified a total of 267 significantly asthma or AR –associating loci from 31 GWAS studies and 170 protein coding GWAS‐level risk genes of asthma or AR. Of these about a third had airway epithelial functions in database and literature search. In addition, many genes have been related to immunity functions and in part to neuro‐musculoskeletal and other functions in literature. These functions overlapped and also formed a strong network in pathway analyses. Still it is noteworthy that not all SNPs would be asthma markers themselves, or that each locus may lead to pathogenesis of AR or asthma. In addition, in about half of the protein coding genes the expression figures of databases was not yet available. Thus, further functional experiments would be needed to study their putative role in airways.

There is still scarce GWAS‐level knowledge of CRS phenotype. After our GWAS catalogue search, a GWAS publication of Islandic and English CRS patients and controls showed that a missense variant in *ALOX15* causing p.Thr560Met alteration in arachidonate 15‐lipoxygenase (*15‐LO* ) confers large genome‐wide significant protection against CRSwNP and CRS. p.Thr560Met, carried by 1 in 20 Europeans, was previously shown to cause near total loss of 15‐LO enzymatic activity [138 ]. The authors suggest that the protective effect of this variant is explained by inactivation of 15‐LO´s catalytic activity, which leads to reduced production of pro‐inflammatory mediators in eosinophils and nasal epithelium [138 ].

Since asthma and allergies are multifactorial disorders affected by both genetic and environmental factors and with multiple phenotypes, it seems likely that several genes are involved, each with a minor effect. It is expected that different genetic pathways are involved with varying proportions in different populations. Allergic diseases are also heterogeneous. Clinically, some patients have allergic rhinitis (AR) alone whereas others have AR and asthma (with or without other allergic manifestations). Few patients have asthma alone. Using transcriptomics analyses in European MeDALL birth cohorts and RNA sequencing in Puerto Rican children, AR as a single disease was found to be associated with Toll‐like receptor gene expression (*TLR* ), whereas rhinitis associated with asthma was linked with IL‐5 and IL‐33, confirming that the two diseases are different [139 ].

We show that many risk genes relate to leukocyte immunity or epithelial cell functions. Approximately half the patients with asthma, regardless of the severity of the disease, and majority of AR cases, exhibit Th2 endotype [67 ]. This endotype is characterized by a predominant activation of Th2 cells that produce cytokines such as IL‐4, ‐5, and ‐13 [67 ]. There is increasing evidence of airway barrier´s role in airway diseases, such as AR [17 ]. Although expression profiles in upper and lower airways might differ [140 ], investigations on nasal epithelial interactions might provide additional knowledge for the lower airway inflammation [43, 141 ].

## Therapeutic implications and future prospects

Since many of the identified risk genes for inflammatory airway diseases are airway epithelial or immunity function related, these functions should have an important role in the search for gene‐environmental interactions, biomarkers and future therapeutics. Metabolism and neuro‐musculoskeletal related functions also seem to have a central role in the development of asthma and AR.

With knowledge of important cascades in asthma and AR pathogenesis, as revealed also by these GWASs, it has been possible to develop medications that specifically target the key players of these cascades. In the 2010′s, biologics suppressing type 2 inflammation have become an important tool when tackling severe eosinophilic asthma, and recently also severe CRSwNP, and new substances are also under investigation [142 ].

Asthma endotypes can broadly be divided to type 2 high or type 2 low asthma [143 ]. Type 2 endotype is defined by the presence of Th2‐ and ILC2‐ inflammatory markers and eosinophilia. Type 2 low endotype is not as well defined; it is characterized by the absence of type 2 markers, and by activation of neutrophils, Th1 and/or Th17 cells [143 ]. Some patients, often with severe asthma, have a mixed population of airway granulocytes (eosinophils and neutrophils), and combined type 2 high and low cytokine signatures, such as IL‐17 or IFN‐γ [144 ]. This heterogeneity could explain why fewer genome‐scale asthma loci have been identified in type 2 low asthma than in type 2 high asthma [143 ]. Lack of data also explains why molecules/pathways relevant to type 2 low asthma were discussed less in this review. Nevertheless, there are several mucosal molecules/pathways that are currently under investigation as potential therapeutic targets for type 2 low or mixed type of asthma, these include IL‐6 [145 ] and TLR‐3,4,7 [146 ].

After this study was performed, new GWASs and functional annotation analysis have been published with new interesting asthma loci such as TNF receptor superfamily member 8 (*TNFRSF8* ) [143 ], and Collagen Type XVI Alpha 1 Chain (*COL16A1* ) [147 ], and more studies are to come. This is indeed very important since further functional activity experiments of candidate genes are still needed to understand the dynamic molecular events behind the pathogenesis of airway diseases. In addition, there is a high need in the future to perform GWASs also on CRS, type 2 low asthma, chronic obstructive lung disease, and rare severe airway diseases, in order to broaden our understanding of all inflammatory airway disease subtypes and to discover new pathways/molecules. This knowledge would potentially improve future preventive and therapeutic strategies for inflammatory airway diseases.

## Author contributions

ST‐S provided the study plan with RR, PM, KD and MK. The data was collected and analyzed by ST‐S, TH, KD, MK. ST‐S, AL, ALH and PK performed the literature review and wrote the manuscript. All authors critically reviewed the data analyses and the manuscript. All authors read and approved the final manuscript.

## Funding

The study was supported in part by research grants from Finnish Medical Foundation, the Finnish Society of Allergology and Immunology, the Jane and Aatos Erkko Foundation, the Finnish Cultural Foundation, Hospital District of Helsinki and Uusimaa (TYH2018103, TYH2019322), Paulo Foundation, the Tampere Tuberculosis Foundation, the Väinö and Laina Kivi Foundation, the Finnish ORL‐HNS Foundation.

## Availability of data and materials

All data generated or analyzed during this study are included in this published article and its Additional files 1, 2.

### Ethics approval and consent to participate

Not applicable.

### Consent for publication

Not applicable.

### Competing interests

STS has acted as paid consultant for Mylan Laboratories Ltd., Biomedical systems Ltd., Roche Products Ltd., and Sanofi S.A. All other authors declare no conflicts of interest.

**Abbreviations**
AR: Allergic rhinitis
CRS: Chronic rhinosinusitis
GWAS: Genome wide association study
GO: Gene Ontology
SNP: Single‐nucleotide polymorphism
KEGG: Kyoto Encyclopedia of Genes and Genomes

## Supplementary information

**Supplementary information** accompanies this paper at https://doi.org/10.1186/s13601‐020‐00347‐6.

## Publisher's Note

Springer Nature remains neutral with regard to jurisdictional claims in published maps and institutional affiliations.

## Supporting information

Supplementary information

**Additional file 1: Table S1.** The full Excel file (as a supple file) of the results of ours including also the SNPs with no relevant signs of airway epithelial expression, and the PMIDs. There are several rows and they are based on 1) associated transcript for particular gene 2) allele changes 3) Multiple GWAS‐studies that have shown the same loci. These explain the over 60 000 rows of the dataset. Abbreviations: N/A = not available, Protein_Atlas_Lung_Expression(TPM02) = 0 = TPM less than 1; 1 = 1 < TPM < 100; 2 = TPM 100 or over, Protein_Atlas_Lung_Expression(TPM) = total TPM value, HPA BrProt exp epi = Bronchial epithelial protein expression of immunohistochemical staining figures of The Human Protein Atlas, HPA Br Prot exp = Bronchial protein expression of immunohistochemical staining figures of The Human Protein Atlas, HPA Nasoph Prot exp epi = Nasopharyngeal epithelial protein expression of immunohistochemical staining figures of The Human Protein Atlas, HPA Nasoph Prot exp = Nasopharyngeal protein expression of immunohistochemical staining figures of The Human Protein Atlas, HPA Nasoph GTEx (RPKM) = The Genotype‐Tissue Expression (GTEx) of Nasopharyngeal mucosa of The Human Protein Atlas, RPKM = Reads Per Kilobase of transcript, per Million mapped reads, HPA Bronch GTEx (RPKM) = The Genotype‐Tissue Expression (GTEx) of Bronchial mucosa of The Human Protein Atlas, GXA = Gene Expression Database.

**Additional file 2: Table S2.** Integrated data from GWAS catalog on asthma, allergy/AR and CRS ‐associating SNPs, the corresponding proteins of which have evidence of human airway epithelial expression from manual literature search. The list of 170 asthma/AR –relevant coding gene names were identified based on GWAS search shown in Fig. 1. We detected that 145/170 genes have reported epithelial gene and/or protein expression based on systematic Human Protein Atlas (HPA) search. We detected that 86/170 genes have at least slight evidence of airway epithelial expression based on literature search, the citations are shown in the list. Abbreviations: Protein_Atlas_Lung_Expression(TPM02) = 0 = TPM less than 1; 1 = 1 < TPM < 100; 2 = TPM 100 or over, Protein_Atlas_Lung_Expression(TPM) = total TPM value, HPA BrProt exp epi = Bronchial epithelial protein expression of immunohistochemical staining figures of The Human Protein Atlas, HPA Br Prot exp = Bronchial protein expression of immunohistochemical staining figures of The Human Protein Atlas, HPA Nasoph Prot exp epi = Nasopharyngeal epithelial protein expression of immunohistochemical staining figures of The Human Protein Atlas, HPA Nasoph Prot exp = Nasopharyngeal protein expression of immunohistochemical staining figures of The Human Protein Atlas, HPA Nasoph GTEx (RPKM) = The Genotype‐Tissue Expression (GTEx) of Nasopharyngeal mucosa of The Human Protein Atlas, RPKM = Reads Per Kilobase of transcript, per Million mapped reads, HPA Bronch GTEx (RPKM) = The Genotype‐Tissue Expression (GTEx) of Bronchial mucosa of The Human Protein Atlas, GXA = Gene Expression Database, Literat_Resp_epith = Literature search result of Airway expression: 0 = no/not known; 1 = some evidence; 2 = evidence exists, Literat Ref (PMID) = PubMed ID for the Literature search result.
